# Forkhead box E1, frequently downregulted by promoter methylation, inhibits colorectal cancer cell growth and migration

**DOI:** 10.1186/s12935-024-03352-y

**Published:** 2024-05-11

**Authors:** Qinlan Shi, Zhongting Huang, Yeye Kuang, Chan Wang, Xiao Fang, Xiaotong Hu

**Affiliations:** 1 Biomedical Research Center and Key Laboratory of Biotherapy of Zhejiang Province, Sir Run Run Shaw Hospital, Zhejiang University, Hangzhou, 310016 China; 2 Department of Anesthesiology, Sir Run Shaw Hospital,, Zhejiang University, Hangzhou, China; 3 Department of Pathology, Sir Run Run Shaw Hospital,, Zhejiang University, Hangzhou, 310016 Zhejiang China

**Keywords:** FOXE1, Methylation, Colorectal cancer, Lymph node metastasis

## Abstract

Forkhead box E1 (*FOXE1*), also known as thyroid transcription factor 2 (*TTF-2*), belongs to a large family of forkhead transcription factors. It plays important roles in embryogenesis, cell growth, and differentiation. Cancer-specific *FOXE1* hypermethylation events have been identified in several cancers. However, the expression and function of *FOXE1* in the tumorigenesis of colorectal cancer remain still unknown. In this study, we examined FOXE1 expression and methylation in normal colon mucosa, colorectal cancer (CRC) cell lines, and primary tumors by immunohistochemistry, semi-quantitative RT-PCR, methylation-specific PCR, and bisulfite genomic sequencing. We found that *FOXE1* was frequently methylated and silenced in CRC cell lines and was downregulated in CRC tissues compared with paired adjacent non-tumor tissues. Meanwhile, low FOXE1 expression was significantly correlated with lymph node metastasis and advanced TNM stages, indicating its potential as a tumor marker. Subsequently, we established colon cancer cell lines with stable FOXE1 expression to observe the biological effect on colorectal cancer, including cell growth, migration, actin cytoskeleton, and growth of human colorectal xenografts in nude mice. Ectopic expression of FOXE1 could suppress tumor cell growth and migration and affect the organization of the actin cytoskeleton together with suppressing tumorigenicity in vivo*. FOXE1* methylation was frequently seen in association with a complete absence of or downregulated gene expression, and *FOXE1* plays a suppressive role in the development and progression of colorectal cancer.

**Supplementary Information:**

The online version contains supplementary material available at 10.1186/s12935-024-03352-y.

## Introduction

Colorectal cancer (CRC) is the third-most common malignant tumor in the world, with more than 1.2 million new cases reported each year. While the incidence of CRC has stabilized in developed countries [1, 2], it has increased in China in recent decades [3]. Although certain progress has been made in the treatment of CRC in recent years, especially new targeted drugs that have improved the survival time of patients with advanced CRC [4], the overall five-year survival rate is still only 30%–60% [5]. The main factor affecting the prognosis of CRC patients is that most patients are already in an advanced stage when diagnosed. Therefore, early diagnosis and treatment are keys to improving the survival rate of CRC patients.

Lifestyle, environment, and diet factors may cause epigenetic changes in disease-related genetic information, which plays an important role in the initiation and progression of CRC [6]. There are many mechanisms of epigenetic modification, including DNA methylation, histone modification, and non-coding RNA regulation [7]. Abnormal DNA methylation is characterized by widespread genome-wide hypomethylation and hypermethylation of CpG islands in the gene-promoter region [8]. Hypomethylation can promote chromosome instability and aneuploidy abnormalities. Hypermethylation of promoters of some tumor suppressor genes often leads to gene silencing, which can lead to the activation or inhibition of various signaling pathways, thus affecting various stages of tumor initiation and progression [9, 10].

Hypermethylation of CpG islands in the promoter regions of genes expressed in normal colon mucosa leads to transcriptional repression in CRC [11, 12]. We can facilitate early diagnosis and prognosis analysis of tumors by analyzing the DNA methylation status of patients [13]. *FOXE1*, also known as thyroid transcription factor 2, is a member of the FOX transcription factor superfamily. The human *FOXE1* gene, located on chromosome 9q22, is 3.5 kb in length and has a single exon [14, 15]. Its complementary DNA encodes a protein consisting of 376 amino acid residues. The FOXE1 protein consists of a FOX domain with nuclear localization signals at both ends and a poly-alanine channel [15] whose length varies in the population from 11 to 19 amino acid residues, with 14 amino acid residues being the most common [16–18].

*FOXE1* is a thyroid-specific transcription factor with an important role in the early stage of thyroid embryo development that regulates the correct migration of thyroid primordia and the normal differentiation and proliferation of thyroid cells. Recent studies have found that FOXE1 is closely related to the occurrence and development of many human tumors, including thyroid cancer [19, 20], pancreatic cancer [21], skin cancer [22], and breast cancer [23]. In the study of skin squamous cell carcinoma, Venza et al. found that the methylation rate of FOXE1 promoter reached 55%, which was significantly higher than that in normal tissues and peripheral blood [17]. Furthermore, FOXE1 expression can be restored following treatment with demethylation reagent 5-Aza [24].

Previously we searched for methylated tumor suppressor genes (TSGs) candidates in digestive cancers through epigenomic (CpG methylome) study and expression profiling of a paired colon cancer cell line (HCT116 and HCT116-DKO with double knock-out of DNMT1 and DNMT3B [25, 26]), and found a significant signal enrichment of CpG methylation at the *FOXE1* promoter in HCT116, but not in DKO, suggesting that *FOXE1* is methylated target in colorectal cancer. However, until now the mechanism of action of FOXE1 in colorectal cancer is not clear. Therefore, in this study, we examined the frequency of *FOXE1* inactivation and explored its functions and mechanisms in CRC.

## Materials and methods

### Cell lines, tumor samples, and normal control tissues

Eleven colorectal cell lines (Colo320, DLD-1, HCE8693, HCT-116, HT-29, RKO, SW620, SW480, LOVO, Colo205, and HCT-8) were used. The cell lines were maintained at 37 °C in a humidified 5% CO_2_ incubator in RPMI 1640, Dulbecco’s modified Eagle’s medium, or McCoy’s5A medium (Gibco BRL, Rockville, MD, USA) supplemented with 10% fetal bovine serum (FBS).

Human CRC tissue was collected from patients with adenocarcinoma confirmed pathologically without direct surgical resection of neoadjuvant therapy between December 2000 and April 2007 in Sir Run Run Shaw Hospital, Zhejiang University School of Medicine, and obtained with informed consent from patients.

FOXE1 immunohistochemical analysis was performed on 10 normal colonic mucosa specimens (paired adjacent non-tumor tissues), 128 primary CRC specimens, 27 metastatic lymph node specimens, and 29 colorectal adenomas (including nine tubular adenomas, eight serrated adenomas, and 12 villous adenomas). Methylation-specific PCR (MSP) testing was performed on the tumor tissue and normal adjacent tissue samples of 35 CRC patients stored in a tissue bank. TNM staging for each patient was based on the seventh edition of tumor TNM staging issued by the American Cancer Federation (AJCC, 2010). In terms of the degree of differentiation, we divided tumors into a highly differentiated group (including highly differentiated tubular adenocarcinoma and papillary adenocarcinoma cases), moderately differentiated group (including moderately differentiated tubular adenocarcinoma cases), and poorly differentiated group (including poorly differentiated adenocarcinoma, undifferentiated adenocarcinoma, mucinous adenocarcinoma, and signed-ring cell carcinoma cases).

### RNA extraction and semi-quantitative RT-PCR

Total RNA was extracted using Trizol reagent (Invitrogen, Carlsbad, CA, USA), as described by the manufacturer. Reverse transcription of the reaction mixture (20 μL) containing total RNA (1 μg) to cDNA was done with M-MLV (Promega Corporation, Madison, WI, USA). The mRNA expression levels of *FOXE1* were determined by semi-quantitative reverse-transcription PCR (RT-PCR) with GoTaq polymerase (Promega Corporation, Madison, WI, USA). The transcription of the principal gene *GAPDH* was used as the internal control. Specific primers were designed according to the *FOXE1* sequence. All sequences of primers used were showed in Table S1.

### Bisulfite treatment and promoter methylation analysis

Genome DNA was extracted from tissues using a Tiangen DNA mini kit (Tiangen, Beijing, China), following the manufac turer’s instructions. Bisulfite modification of DNA was performed as previously described [27]. MSP was performed using AmpliTaq-Gold DNA polymerase (Applied Biosystems, Waltham, MA, USA). The PCR products were identified on 1.5% agarose gels.

### Demethylation treatment using 5-aza-2′-deoxycytidine and trichostatin A

*FOXE1*-silenced cell lines were cultured in a 10-cm dish with 1 × 10^6^ cells each. After overnight culturing, the cells were treated with 5-aza (10 μmol/L) for 72 h and TSA (300 nmol/L) for 24 h. Finally, we harvested the treated cells and extracted DNA and RNA for use.

### Immunohistochemistry

IHC was performed using the ChemMate EnVision detection kit (Dako, Carpinteria, CA, USA) as described by the manufacturer. Briefly, the selected sections were incubated with primary FOXE1 antibody (1:250; Abcam), GAPDH antibody (1: 200; Invitrogen) overnight at 4 °C, and then incubated with ChemMate EnVision/HRP, rabbit/mouse reagent as a secondary antibody. Afterward, the sections were developed using ChemMate DAB + chromogen and counterstained with hematoxylin.The percentage of positive cells was evaluated and scored as 0 (< 5%, negative), 1 (5%–25%, sporadic), 2 (26%–50%, local), 3 (51%–75%, diffuse), or 4 (> 75%, positive) points. The intensity of staining was evaluated and scored as 0 (no staining), 1 (weak staining), 2 (moderate staining), or 3 (strong staining) points. Then, both scores were multiplied to produce an immunoreactivity score (IRS) value ranging from 0 to 12 points to evaluate the association of FOXE1 expression with clinicopathological parameters in a manner corresponding to four expression intensities: 0–1 points, negative; 2–4 points, weakly positive ( +); 5–8 points, moderately positive (+ +); and 9–12 points, strongly positive, (+ + +). Patients were then grouped into two categories based on expression intensity: low expression (negative or weakly positive) and high expression (moderately or strongly positive).

### Immunofluorescence

For immunofluorescence, cells (2 × 10^5^ cells/well) were seeded in the coverslip in a six-well plate with a coverslip inside. After 24 h of culture, cells were fixed in 3.7% paraformaldehyde for 10 min and incubated in PBS with 0.1% Triton X-100 for 4 min on ice, then blocked in 5% FBS for 20 min. Coverslips were moved to a slide and cells were washed three times with PBS; then, we added 200 μL rhodamine phalloidin(Invitrogen, Carlsbad, CA, USA) of 100 nM and additionally incubated cells at room temperature shielded from light for 30 min. Nuclei were stained with PBS with 2 μg/mL of DAPI(Roche, CH) for 10 min. Staining was photographed by an Olympus BX51 microscope (Olympus Corporation, Tokyo, Japan). All of the experiments were repeated three times.

### Construction of FOXE1 expression vector

The DLD-1 CRC cell line was transfected with control or *FOXE1*-expressing plasmid (pCMV6 Entry; Origene, Rockville, MD, USA) using the MegaTran 1.0 transfection reagent (Origene). Stable FOX E1-expressing clones were selected for further study.

### Monolayer and soft agar colony formation assays

1000 FOXE1 expression vector stable transfected and parental cells were plated in 10 cm dishes, respectively, and allowed to grow for two weeks at 37 °C in 5% CO_2_. Surviving colonies (≥ 50 cells/colony) were counted under a microscope after gentian violet staining. The experiments were performed in triplicate.

For the soft agar assay, transfected cells were suspended in a growth medium containing 0.3% agar and seeded into a six-well plate overlaid with 0.5% low-melt agar. Surviving colonies (≥ 50 cells) were photographed and counted after 14–25 days at 37 °C in 5% CO_2_. The experiments were performed in three wells in triplicate.

### Wound-healing assay

Cell motility was assessed using a scratch wound assay. The transfected cells and controls were cultured in six-well dishes until confluent. The cell layers were carefully wounded using sterile tips and washed twice with phosphate-buffered saline. Cells were incubated with fresh medium and photographed under a phase contrast microscope at 0, 12, 24, 36, and 48 h after wounding. The experiments were performed in triplicate.

### Cell migration assay

For the Transwell assay, cells were trypsinized and resuspended in a corresponding medium containing 1% FBS at a density of 1 × 10^6^ cells/mL. One hundred microliters of cell suspension was added to the upper chamber of a Transwell system (Corning, Corning, NY, USA) consisting of inserts containing 8-mm pore-size PET membranes. Six hundred microliters of medium containing 2.5% FBS was placed in the lower chamber. After the indicated amount of incubation time at 37 °C in 5% CO_2_, cells remaining in the upper chamber were removed carefully by cotton swab, and those on the bottom side of the chamber membrane were fixed, stained with 0.25% crystal violet, photographed, and counted under a light microscope. The experiments were performed in triplicate.

### In vivo* subcutaneous tumor model*

All of the in vivo experimental protocols were approved by the animal care committee of Sir Run Run Shaw Hospital, Zhejiang University. Viable *FOXE1*-transfected cells and controls (5 × 10^6^ cells in 0.1 mL of PBS) were injected subcutaneously into the right dorsal flank of six-week-old male BALB/c nude mice (six mice per group). Tumor volume was assessed every two days for five weeks. Tumor volume was calculated with the following formula: (short diameter)^2^ × (long diameter)/2.

### Statistical analysis

Statistical calculations were performed using SPSS version 18.0 for Windows (IBM Corporation, Armonk, NY, USA). Pearson’s chi-squared test was used to analyze the association between FOXE1 expression level and clinicopathological parameters. Results are presented as mean ± standard deviation values, and comparisons between groups were completed by analysis of variance. *P* < 0.05 was considered statistically significant.

## Results

### Methylation and silencing of FOXE1 in CRC cell lines

**Fig. 1:**
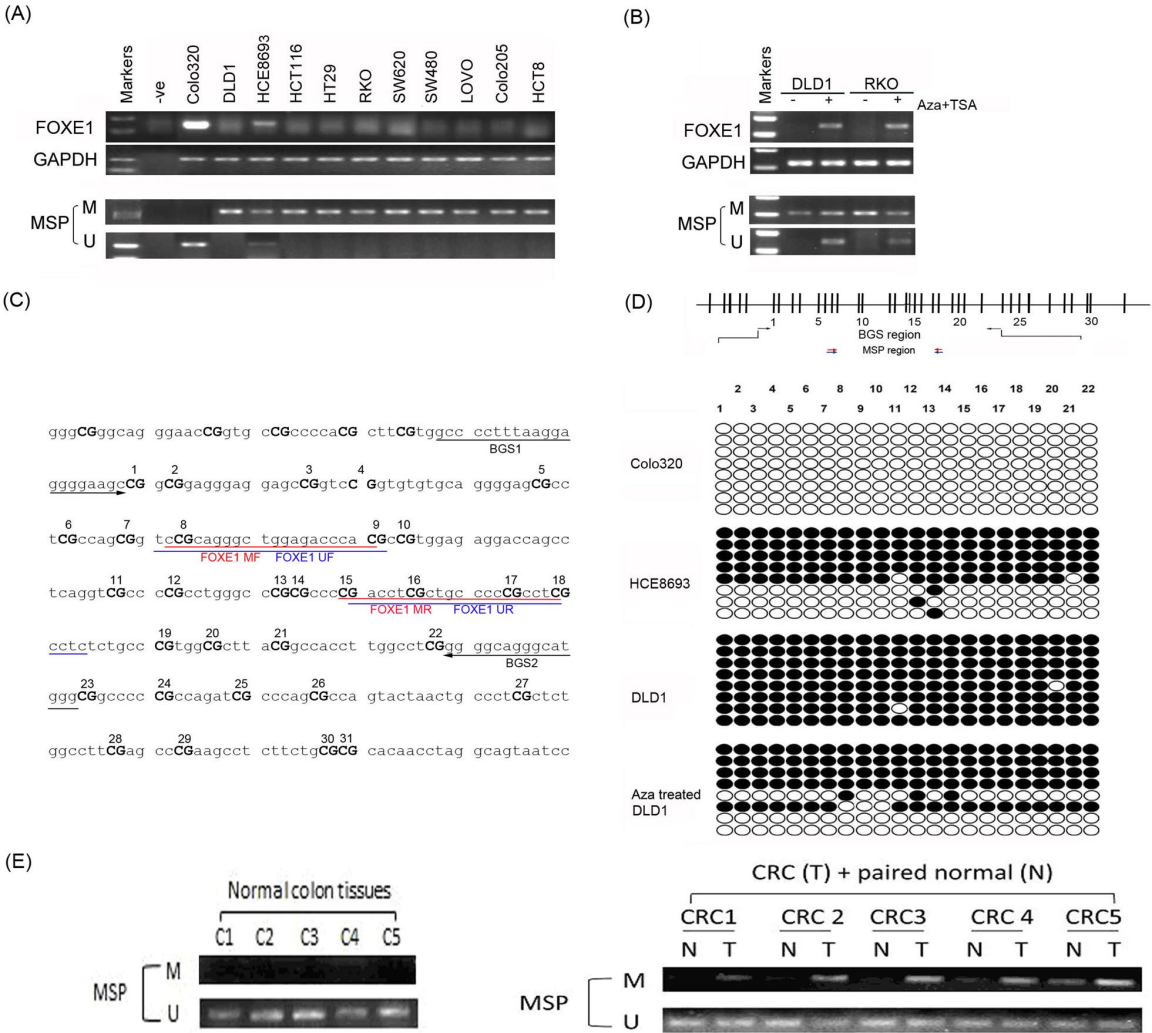
FOXE1 expression and its promoter CGI methylation in CRC cell lines and primary tumors. **A** FOXE1 was greatly reduced or silenced in most colorectal cancer cell lines (9/11). **B** Pharmacological demethylation with 5-Aza and histone deacetylase inhibitor TSA restored FOXE1 expression in methylated and silenced cell lines and demethylation was confirmed by MSP. **C** Sequence of promoter region CpG island. **D** Typical BGS results. Each short vertical line is one CpG site. FOXE1 methylation was analyzed by BGS analysis. One row of circles represents an individual allele of the FOXE1 promoter analyzed. Each circle represents one CpG site and filled circles are methylated CpG sites. **E** Representative MSP results of primary tumor tissues (T) and their paired normal tissues (N) and MSP results of normal colorectal mucosa biopsy tissues

First, we tested FOXE1 expression in CRC cell lines by semi-quantitative RT-PCR. Our results showed that FOXE1 was expressed in only two cell lines (Colo320 and HCE8693), while being silenced in the other nine. *FOXE1* methylation status was tested by MSP to elucidate the effect of promoter methylation in the downregulation of *FOXE1*. Methylation of *FOXE1* was detected in all of the cell lines with silenced *FOXE1* expression (Fig. 1A). We treated two FOXE1 expression silenced CRC cell lines (DLD-1 and RKO) with 5-Aza and TSA and found that FOXE1 expression could be restored. Representative results are shown in Fig. 1B.

We further examined the detailed methylation profiles of *FOXE1* CGI by bisulfite genomic sequencing analysis, including those CpG sites analyzed by MSP (Fig. 1C). The results showed that methylated CpG sites could not be found in the Colo 320 cell line with high FOXE1 expression, while it was markedly increased in the HCE8693 cell line with low FOXE1 expression and densely detected in the DLD-1 cell line with *FOXE1* silencing. The representative results are shown in Fig. 1D.

Meanwhile, both MSP and bisulfite genomic sequencing showed that *FOXE1* CGI was remarkably demethylated after treatment with 5-Aza and TSA (Fig. 1B, D). These results suggest a direct link between CGI methylation and FOXE1 expression.

We also analyzed *FOXE1* methylation status in 10 normal colorectal tissues, 35 primary colorectal tumors, and paired adjacent non-tumor tissues. *FOXE1* methylation was not detected in normal mucosal tissues but was detected in 85.7% (30/35) of tumors and only 20.0% (7/35) of adjacent non-tumor tissues. Representative results are shown in Fig. 1E. These results suggested that *FOXE1* gene methylation may be closely related to the development of CRC.

### FOXE1 was downregulated in CRC tissues

We investigated FOXE1 expression in a total of 128 primary CRC tissues, 27 metastatic lymph node tissues, 29 colorectal adenomas (tubular adenoma, serrated adenoma, and villous adenoma) tissues, and 10 normal colorectal mucosal tissues by immunohistochemistry. High FOXE1 expression was shown in all of the normal mucosal epithelium (10/10) tissues but only 25% (32/128) of primary CRC tissues and 7.4% (2/27) of metastatic lymph node tissues. This trend suggests that the expression of FOXE1 in CRC is significantly decreased, and decreased expression of FOXE1 may be associated with lymph node metastasis of CRC (Table 1).

**Table 1:** FOXE1 expression in normal colon mucosa and colorectal cancer tissues

		FOXE1 immunostaining		*P*-value
Tissue samples	n	Low (%)	High (%)	
Normal colonic mucosasamples Pramary colorectal cancer Metastatic lymph nodes Tubular colorectal adenoma Serrated colorectal adenoma Villous colorectal adenoma	10	0 (0)	10 (100)	0.000*
	128 27 9 8 12	96 (75.0) 25 (92.6) 2 (22.2) 0 (0) 8 (66.7)	32 (25.0) 2 (7.4) 7 (77.8) 8 (100) 4 (33.3)	0.006*

**Fig. 2:**
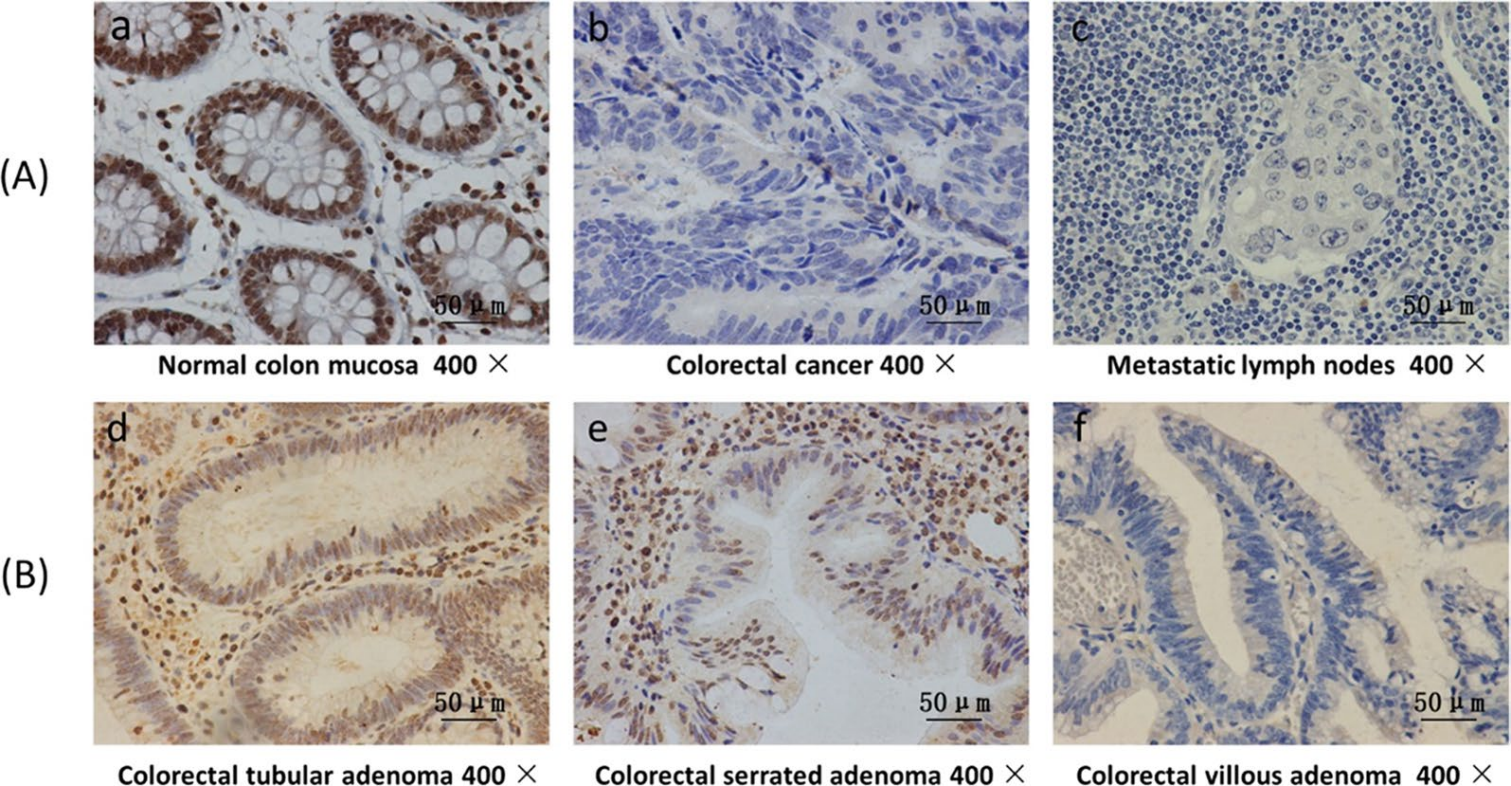
Representative immunohistochemical staining of FOXE1, which was strongly expressed in the cytoplasm and nucleus of normal epithelial and some stromal cells but silenced or weakly expressed in tumor cells and lymph nodes, moreover, FOXE1 was highly expressed in colorectal tubular adenoma and serrated adenoma but weakly expressed in villous adenoma: normal colon mucosa (**a**), primary colorectal cancer (**b**), metastatic lymph node (**c**), colorectal tubular adenoma (**d**), serrated adenoma (**e**), and villous adenoma (**f**)

Interestingly, the high expression rate of FOXE1 in tubular adenoma and serrated adenoma was like that found in the normal colon mucosa, while FOXE1 expression in villous adenoma was significantly decreased, close to the level of that associated with primary CRC. Representative immunohistochemical staining results are shown in Fig. 2.

### Correlation between FOXE1 expression and clinicopathological features of CRC patients

We analyzed the correlation between FOXE1 protein expression and the clinicopathological features of CRC patients. The tissues were separated into two groups by immunohistochemistry result: low expression (negative or weakly positive) and high expression (moderately or strongly positive). The correlation between FOXE1 protein-expression levels and clinicopathological features of CRC patients, including sex, age, tumor location, histological differentiation, depth of invasion, lymph node and distant metastasis, and TNM stage, is shown in Table 2. The results suggest that the expression of FOXE1 protein in primary CRC is significantly correlated with lymph node metastasis and TNM stage.

**Table 2:** Correlation of FOXE1 protein expression and clinicopathological features in 128 cases of primary colorectal cancer

		FOXE1 immunoreactivity		*P*-value
	n	Low expression (%)	High expression (%)	
Total	128	96 (75.0)	32 (25.0)	
Gender				
Male	64	50 (78.1)	14 (21.9)	0.414
Female	64	46 (71.9)	18 (28.1)	
Age				
Median	60.15			0.307
≥ 60.15	62	44 (71.0)	18 (29.0)	
< 60.15	66	52 (78.8)	14 (21.2)	
Location				
Rectum	78	64 (82.1)	14 (17.9)	0.066
Left colon	29	18 (62.1)	11 (37.9)	
Right colon	21	14 (66.7)	7 (33.3)	
Histopathological grade				
Well	46	34 (73.9)	12 (26.1)	0.735
Moderately	47	37 (78.7)	10 (21.3)	
Poorly	35	25 (71.4)	10 (28.6)	
pT				
pT1	3	1 (33.3)	2 (66.7)	0.216
pT2	29	23 (79.3)	6 (20.7)	
pT3	92	70 (76.1)	22 (23.9)	
pT4	4	2 (50.0)	2 (50.0)	
pN				
pN0	60	38 (63.3)	22 (36.7)	0.004*
pN1/2	68	58 (85.3)	10 (14.7)	
pM				
pM0	113	84 (74.3)	29 (25.7)	0.634
pM1	15	12 (80.0)	3 (20.0)	
Stage				
I/ II	56	36 (64.3)	20 (35.7)	0.014*
III/ IV	72	60 (83.3)	12 (16.7)	

* Statistically significant (*p* < 0.05)

Interestingly, the high expression rate of FOXE1 in tubular adenoma and serrated adenoma was like that measured in normal colon mucosal tissues, while the FOXE1 expression level in villous adenoma was significantly decreased, being closer to the level in primary CRC.

### FOXE1 expression inhibited CRC cell proliferation and migration

**Fig. 3:**
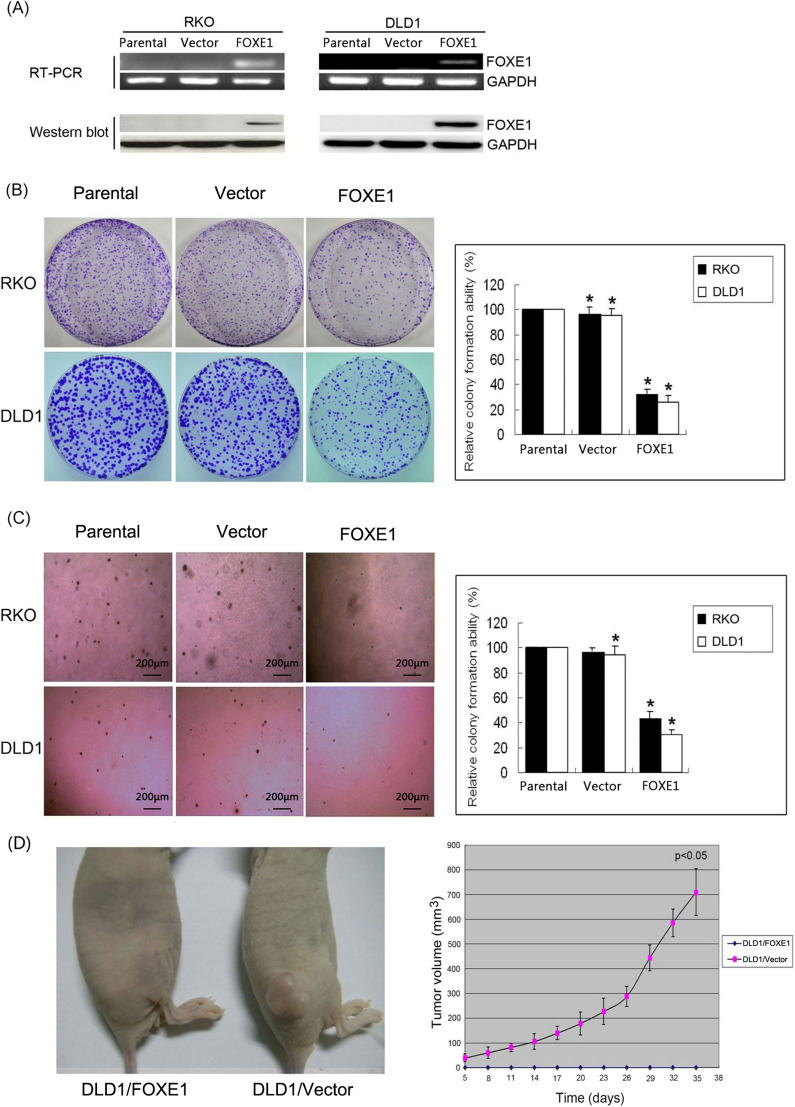
FOXE1 expression inhibited proliferation and migration of CRC cells in vitro an in vivo. **A** FOXE1 expression in stably transfected cells as confirmed by RT–PCR and western blotting. **B** Monolayer culture and quantitative analysis of colony number; values are presented as the mean ± SD. **C** Soft agar assay and quantitative analysis of colony number; values are presented as the mean ± SD. **D** Images of xenograft tumors formed in nude mice injected with FOXE1-Transfected DLD-1 cells (Left) and empty vector-transfected DLD-1 cells(right), and tumor growth curves of DLD-1 cells

To investigate the biological function of FOXE1, a FOXE1-expressing plasmid was stably transfected into DLD-1 and RKO cells without FOXE1 expression. Then, ectopic expression of FOXE1 was confirmed by RT-PCR and western blotting (Fig. 3A).

**Fig. 4:**
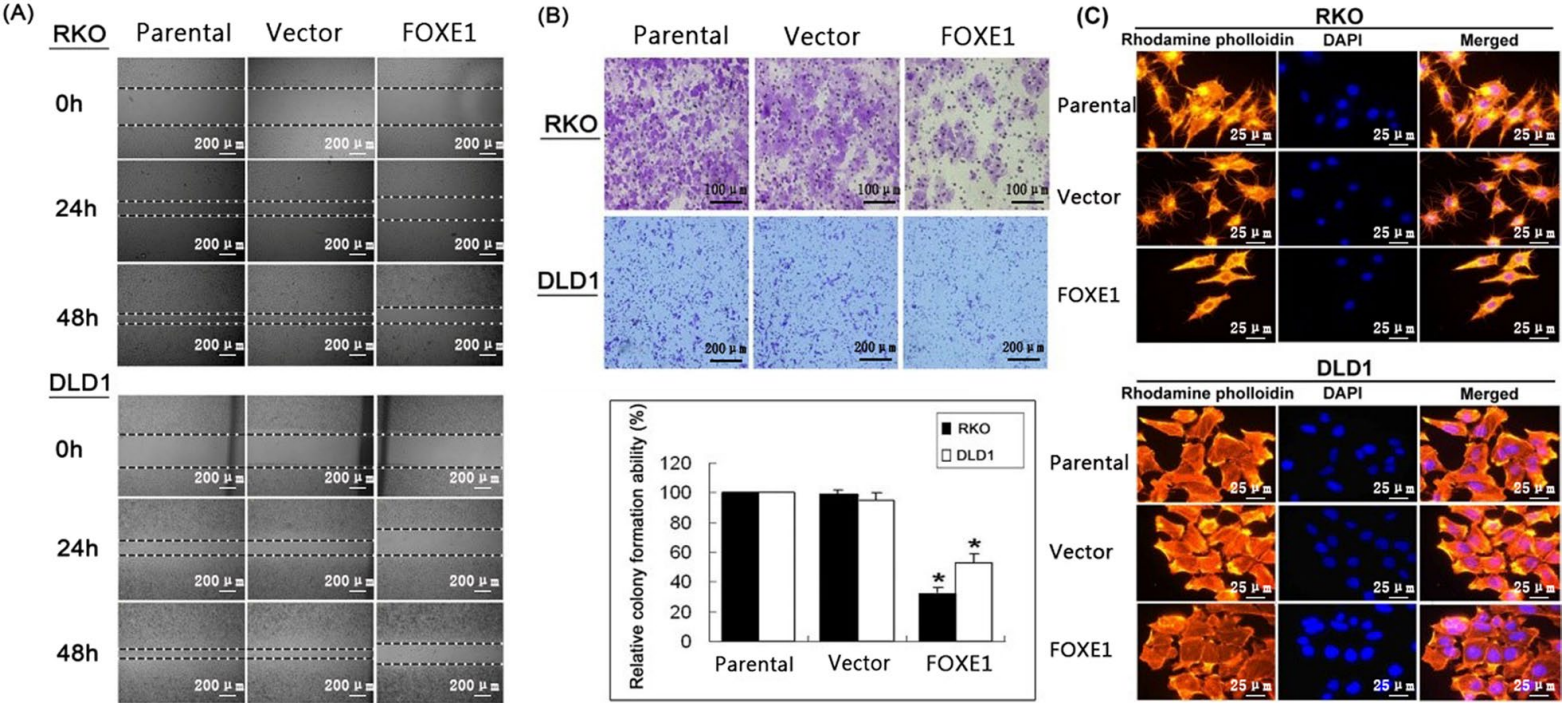
FOXE1 expression inhibited migration of CRC cells. **A** Representative results of wound-healing assay. **B** Transwell migration assay and quantitative analysis of relative cell count shown; values are presented as mean ± SD (*P < 0.05) **C** Effect of FOXE1 transfection on morphology of DLD-1 and RKO cells, rhodamine-labeled Phalloidin was used to indicate actin polymerization

To evaluate the effects of FOXE1 expression on the migration of colorectal tumor cells, wound-healing and Transwell migration assays were performed. Wound healing was prolonged in the *FOXE1*-transfected cells compared with that in the control cells (Fig. 4A). *FOXE1*-transfected cells also showed significant suppression of migration compared with that in control cells after 24 h of incubation during Transwell assay (Fig. 4B).

Moreover, both monolayer culture and soft agar assays showed that the colonies formed among the *FOXE1*-transfected cells were significantly fewer in number and smaller in size than those of the control cells (*P* < 0.05, Fig. 3B, C).

To observe the morphology of DLD-1 cells, cells transfected with *FOXE1* or empty vector and parental cells were stained with rhodamine–phalloidin to observe the organization of actin fibers. Cells transfected with *FOXE1* displayed diffuse cytoplasmic actin arranged irregularly and significantly fewer thin stress fibers, whereas control groups of cells Showed better arrangement of actin fibers, supporting a possible regulatory role for FOXE1 in actin cytoskeleton reorganization (Fig. 4C).

These results indicate that FOXE1 possesses growth and migration-inhibitory activities in CRC cells and might function as a tumor suppressor.

### FOXE1 inhibited CRC cell growth in nude mice

Finally, we investigated whether FOXE1 expression had a suppressive effect on tumor cell growth in vivo. We modeled xenografts in nude mice using DLD-1/vector-transfected and DLD-1/*FOXE1*-transfected cells. All six of the xenografts of DLD-1/vector-transfected cells showed tumor growth. Overall, the tumor formation rate was 100%, and the tumor volume reached 709 ± 95 mm^3^ after five weeks. The growth curve of a transplanted tumor is shown in Fig. 3D. However, there was no obvious subcutaneous tumor found among the six nude mice injected with DLD-1/*FOXE1*-transfected cells, and the tumor-formation rate was 0%. Our results suggest that FOXE1 can significantly inhibit tumorigenesis in vivo.

## Discussion

FOXE1 has been widely studied as a thyroid-specific transcription factor in thyroid cancer, and its polymorphisms (rs965513 and rs1867277) are thought to increase susceptibility to thyroid cancer [28]. In recent years, the promoter region of FOXE1 has been found to be methylated in various tumors. Venza et al. reported that the loss of or down-regulation of FOXE1 expression in cutaneous squamous cell carcinoma is related to hypermethylation of the promoter region of FOXE1 [24]. This suggests that epigenetic mechanisms such as DNA methylation may be the main reason for the silencing of FOXE1 expression in tumors. FOXE1, as a tumor-specific methylation marker, can be detected in the pancreatic juice of pancreatic cancer patients [21] and serum of breast cancer patients [23]. Melotte et al. discovered that high expression of FOXE1 can reduce the number of clones in CRC cell lines [29]. CRC arising on the background of inflammatory bowel disease (IBD), colitis-associated cancer (CAC), is often more aggressive than sporadic CRC [30]. Low expression of FOXE1 has been shown to be positively associated with inflammatory bowel disease (IBD) [31].This suggests its potential value as a tumor marker in clinical diagnosis.

In this study, we found that FOXE1 mRNA was not expressed in most CRC cell lines by RT-PCR. However, FOXE1 gene-promoter hypermethylation was confirmed in these tumor cell lines. After imbuing tumor cells with silenced or reduced expression of FOXE1 using demethylation reagent 5-AZA and histone deacetylase inhibitor TSA, we found that FOXE1 expression could be restored, indicating that FOXE1 methylation is the main cause of FOXE1 gene-silencing. FOXE1 is methylated in most CRC tissues but not in normal adult colorectal mucosal tissues. Unlike in CRC cell lines, non-methylated mRNA fragments were also detected in primary CRC tissues, which may be related to the presence of non-tumor cells in primary tumor tissues. Similar results were also found in the study of Zhang et al., who found that the methylation rate was 63.2% in 19 stage I CRC specimens, while no methylation was found in normal intestinal mucosal epithelium samples.

These results suggest that methylation of the FOXE1 promoter region is a frequent and tumor-specific molecular event in CRC. Immunohistochemistry was used to detect the expression of FOXE1 protein in CRC tissues. The results showed that the expression of FOXE1 protein in primary CRC tissues was significantly lower than that in normal colon mucosa tissues, and the expression of FOXE1 protein was highly expressed in all 10 of the normal colon mucosal samples. The expression of FOXE1 in metastatic lymph nodes was also significantly lower than that in primary CRC tissues. Correlation analysis between FOXE1 expression and clinicopathological features showed that loss of FOXE1 expression is significantly associated with lymph node metastasis and poor TNM stage in CRC patients. These results suggest that the downregulation of FOXE1 expression may be associated with the progression of CRC.

In the 1970s, Morson et al. proposed the classic sequential theory of normal mucosa, benign adenoma, and adenocarcinoma in CRC [32]. The evolution of adenoma to adenocarcinoma is associated with genomic and epigenetic alterations that accumulate progressively during the development of CRC. It has been reported that more than 90% of colorectal tumors are derived from adenomas through this classical pathway [33]. Colorectal adenoma is considered a precancerous lesion preceding CRC. In terms of pathological features of colorectal adenoma, villous colorectal adenoma has the highest malignant tendency, while tubular colorectal adenoma and serrated colorectal adenoma are significantly less likely to develop into cancer. Our immunohistochemical results showed that the positive rate of FOXE1 protein in villous colorectal adenomas of the large intestine was significantly lower than that in tubular colorectal adenomas and serrated colorectal adenomas. The difference in FOXE1 protein expression in different types of colorectal adenomas may be related to tumor heterogeneity. Further studies are needed to investigate the role of FOXE1 in the evolution of colorectal adenoma to adenocarcinoma in the future.

Through cell viability assay, monolayer, and soft agar colony formation assays, we found that the growth rate and proliferation ability of tumor cell lines were significantly decreased after being transfected with FOXE1. Although FOXE1 did not promote the apoptosis of tumor cells, it may inhibit the growth of tumor cells by arresting the cell cycle. Consistent with the results of FOXE1 inhibition of tumor cell proliferation in vitro, FOXE1 transfection could significantly inhibit the tumorigenesis of DLD-1 cells in vivo. We also found that the FOXE1 gene had a certain inhibitory effect on migration compared relative to the trend in the control group, which is also consistent with the immunohistochemical results.

The cellular actin skeleton plays an important role in maintaining cell morphology and cell motility. Actin comes in monomeric and polymeric forms, with the monomeric form of actin, known as G-actin, being globular, and the polymeric form of actin, called F-actin, being fibrous. The polymerization and reorganization of the cellular actin skeleton are the first and key steps of cell migration. During cell migration, the content of F-actin is often increased (actin polymerization), which enhances cell motility [34]. In our experiment, we labeled F-actin with rhodamine–phalloidin and carried out cellular immunofluorescence assays. The results suggested that FOXE1 could prevent the recombination of F-actin and significantly reduce the number of stress fibers, which may be a reason why FOXE1 weakens the migration ability of tumor cells.

In conclusion, our results suggest that the silencing or downregulation of FOXE1 in CRC is mainly regulated by methylation of the promoter region, and FOXE1 may play a role as a tumor suppressor gene. Although the specific molecular mechanism of FOXE1 in the tumorigenesis and progression of CRC is still unclear, the hypermethylation of the FOXE1 gene-promoter region may have potential value for the clinical diagnosis of CRC.

### Supplementary Information

Supplementary Materials 1: Table S1. Sequences of the primers for PCR

## Data Availability

The datasets generated during and/or analysed during the current study are available from the corresponding author on reasonable request.

## References

[1] Center MM, Jemal A, Ward E (2009). International trends in colorectal cancer incidence rates. Cancer Epidemiol Biomarkers Prev.

[2] Jemal A, Bray F, Center MM, Ferlay J, Ward E, Forman D (2011). Global cancer statistics. CA Cancer J Clin.

[3] Sung J, Lau J, Kl Goh, Leung WK (2005). Asia pacific working group on colorectal cancer. increasing incidence of colorectal cancer in asia: implications for screening. Lancet Oncol.

[4] Wolpin BM, Mayer RJ (2008). Systemic treatment of colorectal cancer. Gastroenterology.

[5] Sava S, Younghusband HB (2010). dbCPCO: a database of genetic markers tested for their predictive and prognostic value in colorectal cancer. Hum Mutat.

[6] Lao VV, Grady WM (2011). Epigenetics and colorectal cancer. Nat Rev Gastroenterol Hepatol.

[7] Laird PW (2005). Cancer epigenetics. Hum Mol Genet.

[8] Karpiński P, Sasiadek MM, Blin N (2008). Aberrant epigenetic patterns in the etiology of gastrointestinal cancers. J Appl Genet.

[9] Link A, Balaguer F, Shen Y, Lozano JJ, Leung HC, Boland CR, Goel A (2013). Curcumin modulates DNA methylation in colorectal cancer cells. PLoS ONE.

[10] Baylin SB, Jones PA (2011). A decade of exploring the cancer epigenome—biological and translational implications. Nat Rev Cancer.

[11] Migheli F, Iore L (2012). Epigenetics of colorectal cancer. Clin Genet.

[12] Wong JJL, Hawkins NJ, Ward RL (2007). Colorectal cancer: a model for epigenetic tumorigenesis. Gut.

[13] Qureshi SA, Bashir MU, Yaqinuddin A (2010). Utility of DNA methylation markers for diagnosing cancer. Int J Surg.

[14] Katoh M (2004). Human FOX gene family (Review). Int J Oncol.

[15] Chadwick BP, Obermayr F, Frischauf AM (1997). FKHL15, a New human member of the Forkhead gene family located on chromosome 9q22. Genomics.

[16] Hishinuma A, Ohyama Y, Kuribayashi T, Nagakubo N, Namatame T, Shibayama K, Arisaka O, Matsuura N, Ieiri T (2001). Polymorphism of the polyalanine tract of thyroid transcription factor-2 gene in patients with thyroid dysgenesis. Eur J Endocrinol.

[17] Venza M, Visalli M, Venza I, Torino C, Saladino R, Teti D (2006). FOXE1 gene mutation screening by multiplex PCR/DHPLC in CHARGE syndrome and syndromic and non-syndromic cleft palate. J Chromatogr B Analyt Technol Biomed Life Sci.

[18] Carré A, Castanet M, Sura-Trueba S, Szinnai G, Van Vliet G, Trochet D, Amiel J, Léger J, Czernichow P, Scotet V, Polak M (2007). Polymorphic length of FOXE1 alanine stretch: evidence for genetic susceptibility to thyroid dysgenesis. Hum Genet.

[19] Penna-Martinez M, Epp F, Kahles H, Ramos-Lopez E, Hinsch N, Hansmann ML, Selkinski I, Grünwald F, Holzer K, Bechstein WO, Zeuzem S, Vorländer C, Badenhoop K (2013). FOXE1 association with differentiated thyroid cancer and its progression. Thyroid.

[20] Matsuse M, Takahashi M, Mitsutake N, Nishihara E, Hirokawa M, Kawaguchi T, Rogounovitch T, Saenko V, Bychkov A, Suzuki K, Matsuo K, Tajima K, Miyauchi A, Yamada R, Matsuda F, Yamashita S (2011). The FOXE1 and NKX2-1 loci are associated with susceptibility to papillary thyroid carcinoma in the Japanese population. J Med Genet.

[21] Matsubayashi H, Canto M, Sato N, Klein A, Abe T, Yamashita K, Yeo CJ, Kalloo A, Hruban R, Goggins M (2006). DNA methylation alterations in the pancreatic juice of patients with suspected pancreatic disease. Cancer Res.

[22] Venza I, Visalli M, Tripodo B, Lentini M, Teti D, Venza M (2010). Investigation into FOXE1 genetic variations in cutaneous squamous cell carcinoma. Br J Dermatol.

[23] Weisenberger DJ, Trinh BN, Campan M, Sharma S, Long TI, Ananthnarayan S, Liang G, Esteva FJ, Hortobagyi GN, McCormick F, Jones PA, Laird PW (2008). DNA methylation analysis by digital bisulfite genomic sequencing and digital MethyLight. Nucleic Acids Res.

[24] Venza I, Visalli M, Tripodo B, De Grazia G, Loddo S, Teti D, Venza M (2010). FOXE1 is a target for aberrant methylation in cutaneous squamous cell carcinoma. Br J Dermatol.

[25] Wang J, Xia Y, Li L, Gong D, Yao Y, Luo H, Lu H, Yi N, Wu H, Zhang X, Tao Q, Gao F (2013). Double restriction-enzyme digestion improves the coverage and accuracy of genome-wide CpG methylation profiling by reduced representation bisulfite sequencing. BMC Genomics.

[26] Li L, Zhang Y, Fan Y, Sun K, Su X, Du Z, Tsao SW, Loh TKS, Sun H, Chan ATC, Zeng Y, Chan W, Chan FK, Tao Q (2015). Characterization of the nasopharyngeal carcinoma methylome identifies aberrant disruption of key signaling pathways and methylated tumor suppressor genes. Epigenomics.

[27] Ying J, Li H, Seng TJ, Langford C, Srivastava G, Tsao SW, Putti T, Murray P, Chan AT, Tao Q (2006). Functional epigenetics identifies a protocadherin PCDH10 as a candidate tumor suppressor for nasopharyngeal, esophageal and multiple other carcinomas with frequent methylation. Oncogene.

[28] Kimura S (2011). Thyroid-specific transcription factors and their roles in thyroid cancer. J Thyroid Res.

[29] Melotte V, Yi JM, Lentjes MH, Smits KM, Van Neste L, Niessen HE, Wouters KA, Louwagie J, Schuebel KE, Herman JG, Baylin SB, van Criekinge W, Meijer GA, Ahuja N, van Engeland M (2015). Spectrin repeat containing nuclear envelope 1 and forkhead box protein E1 are promising markers for the detection of colorectal cancer in blood. Cancer Prev Res (Phila).

[30] Tine J, Edward VL, Fernando SV, Harmsen WS, Zinsmeister AR, Smyrk TC, Tremaine WJ, Melton LJ, Munkholm P, Sandborn WJ (2006). Incidence and prognosis of colorectal dysplasia in inflammatory bowel disease: a population based study from Olmsted County. Minnesota Inflamm Bowel Dis.

[31] Cinzia P, Joost L, Paolo DR, Grooteclaes M, Coruzzi A, Montana C, Novelli M, Bordi C, de Angelis GL, Bassett P, Bigley J, Warren B, Atkin W, Forbes A (2014). FOXE1 and SYNE1 genes hypermethylation panel as promising biomarker in colitis-associated colorectal neoplasia. Inflamm Bowel Dis.

[32] Hurlstone DP, George R, Brown S (2007). Novel clinical in vivo roles for indigo carmine: high-magnification chromoscopic colonoscopy. Biotech Histochem.

[33] Arends MJ (2013). Pathways of colorectal carcinogenesis. Appl Immunohistochem Mol Morphol.

[34] Pollard TD, Borisy GG (2003). Cellular motility driven by assembly and disassembly of actin filaments. Cell.

