# Supplementary material for: Forkhead box E1, frequently downregulted by promoter methylation, inhibits colorectal cancer cell growth and migration
Source: Cancer Cell Int. 2024 May 11;24:169. doi: 10.1186/s12935-024-03352-y (PMC11088116; doi:10.1186/s12935-024-03352-y)
Supplement: Supplementary file 1 — Supplementary Materials 1: Table S1. Sequences of the primers for PCR [file 12935_2024_3352_MOESM1_ESM.docx]

**Table S1.** **Sequences of the primers for PCR**

| **primers** | **Primer sequences(5’-3’)** |
| --- | --- |
| **For determining *FOXE1* mRNA levels by RT-PCR** | |
| *FOXE1*-F | GACCACGGTGGACTTCTACG |
| *FOXE1*-R | CCCTACGCTGGCTCACAT |
| **For detecting methylated *FOXE1* promoter by MSP** | |
| *FOXE1*-mF | TCGTAGGGTTGGAGATTTAC |
| *FOXE1*-mR | GAAACGAAAACAACGAAATCG |
| **For detecting unmethylated *FOXE1* promoter by MSP** | |
| *FOXE1*-uF | TTTGTAGGGTTGGAGATTTATG |
| *FOXE1*-uR | AAAACAAAACAAAAACAACAAAATC |
| **For detecting detail methylation status of *FOXE1* promoter by BGS** | |
| *FOXE1*-BGS1 | GGTTTTTTTAAGGAGGGGA |
| *FOXE1*-BGS2 | CCCATGCCCTACCCC |
| **For standardizing the amount of RNA for RT-PCR** | |
| *GAPDH*-F | TCCTGTGGCATCCACGAAACT |
| *GAPDH*-R | GAAGCATTTGCGGTGGACGAT |
